# The effect of aging on identification of Mandarin consonants in normal and whisper registers

**DOI:** 10.3389/fpsyg.2022.962242

**Published:** 2022-08-12

**Authors:** Min Xu, Jing Shao, Hongwei Ding, Lan Wang

**Affiliations:** ^1^Institute of Corpus Studies and Applications, Shanghai International Studies University, Shanghai, China; ^2^Department of English Language and Literature, Hong Kong Baptist University, Kowloon Tong, Hong Kong SAR, China; ^3^Speech-Language-Hearing Center, School of Foreign Languages, Shanghai Jiao Tong University, Shanghai, China; ^4^Shenzhen Institutes of Advanced Technology, Chinese Academy of Sciences, Shenzhen, China

**Keywords:** Mandarin, speech perception, consonant, aging, whispering

## Abstract

Consonant perception in older adults has been widely explored in recent years. However, how aging affects the identification of Mandarin consonants, especially in whispered condition, are under studied. Mandarin consonants and whispering speech have unique features, which may result in different identification difficulties for older adults. The current study investigated older adults' identification of Mandarin consonants in phonated and whispered conditions in comparison with the performance of younger adults. It was found that in phonated condition, older adults showed the lowest accuracy for affricatives and fricatives owing to their insensitivity to high-frequency information. The lower accuracy of affricatives and plosives was largely attributed to the difficulty in recognizing articulatory places. Identifying aspirated plosives was much more difficult than unaspirated counterparts for older adults. In whispered condition, the recognition of voiced consonants and aspirated-unaspirated distinction became challenging, especially for older adults. Contrary to the expectation, some consonants became easier to be recognized in the whispered condition, i.e., /p^h^, tɕ^h^, x/. These findings enrich our understanding of how aging affects consonant identification in different languages and less ideal conditions. It also suggests that the listener's ability, language uniqueness, and characteristics of distorted speech should be all taken into consideration when investigating speech perception in adverse conditions.

## Introduction

### The deteriorated abilities in older adults

Aging is accompanied by various declined abilities, such as deteriorated memory and lower capacity for attention and inhibition. Apart from that, one of the most common phenomena in older adults is age-related hearing loss. It has been well-documented that the aging ear is associated with the loss of sensory hair cells (Schuknecht, 1974; Gates and Mills, 2005), which can amplify the traveling wave vibration in the auditory system. The reduced amplification led to high-frequency information decoded in the cochlear base being more likely to be affected than low-frequency information decoded in the cochlear apex (Cooper and Rhode, 1997; Robles and Ruggero, 2001). The insensitivity to high-frequency information in the aging population adversely affected their perception of certain consonants, including fricatives and affricatives. For example, Scharenborg et al. (2015) found that older adults were less sensitive to acoustic differences between fricatives /f/ and /s/ in categorical perception and hearing loss further lowered the sensitivity.

Another well-explored issue with older adults is their poor ability to process temporal information. The evidence was from psychoacoustic experiments, categorical perception using stimuli with varied temporal cues, and temporally distorted speech perception, such as time-compressed, reverberated, and noise-masked speech. The psychoacoustic experiments mainly revealed this deficit through the threshold examination of temporal order discrimination/identification and gap detection (Fitzgibbons and Gordon-Salant, 1998; Grose et al., 2006). With regard to speech perception, most studies modified the duration of VOT, closure duration, and formant transition. It was found that older adults showed inferior ability in temporal processing in speech perception (Ginzel et al., 1982; Gordon Salant et al., 2006). The temporally distorted speech perception was usually related to age-related hearing loss, which suggested that hearing loss played an influential role in temporal information processing (Rooij and Plomp, 1991). However, there were other studies showing that aging alone may also contribute to temporal processing deficits, independent from hearing loss (Fitzgibbons and Gordon-Salant, 1998; Strouse et al., 1998). This temporal processing deficit was also proved by neural evidence (see e.g., Walton, 2010 for a review). Based on these existing findings, it is likely that older adults' temporal processing deficits may negatively affect Mandarin consonant perception.

Apart from the deteriorated abilities mentioned above, aging is also associated with other inferior abilities, such as spectral processing deficit and insensitivity to pitch and intensity, which may also hinder speech perception (He et al., 1998, 2007; Harris et al., 2007; Souza et al., 2011; Bidelman et al., 2014). For example, He et al. (1998) and Harris et al. (2007) reported that older adults had higher thresholds for frequency and intensity than younger adults, although two groups had closely matched audiograms. Older adults also classified vowel continuum more variably which indicated they utilized spectral information less effectively (Bidelman et al., 2014).

### Consonant perception in older adults

In the past few years, a large number of studies have confirmed the adverse influence of aging on consonant perception (Gelfand et al., 1985; Ohde and Abou-Khalil, 2001; Tremblay et al., 2003; Scharenborg et al., 2015; Kalaiah et al., 2016; Fogerty et al., 2017; Rishiq et al., 2020). Some studies employed synthesized continua varied in the transition of second formant, VOT or frication duration to examine the categorical perception of consonants, such as plosives (/b, d, g/; /p, t, k/) and fricatives (/f, s/). This method is thought to reveal older adults' specific abilities in consonant perception. For example, Harkrider et al. (2005) and Tremblay et al. (2003) revealed older adults' temporal processing deficit and their inferior ability to process dynamic spectral information in categorical perception experiments. On the other hand, some studies examined the identification of a larger scope of consonants using natural stimuli. This line of research may better reflect older adults' difficulty in perceiving consonants in their daily communications. For example, identifying places of articulation was most difficult, followed by manners of articulation and voicing (Xia et al., 2018). Furthermore, consonants with nasality, glide/liquid, and sibilance quality were easier to be recognized than stop, frication, and place information (Gelfand et al., 1985).

The above-mentioned studies mostly focused on English. Existing works on the aging effects on phoneme perception in Mandarin mainly focus on the lexical tones and vowels (Yang et al., 2015; Wang et al., 2017a,b; Feng et al., 2019, 2021; Liu et al., 2021), while consonant recognition was understudied. There was only one study that examined the perception of 23 Mandarin consonants [including approximants/lateral approximants (A/LA)] in older adults (Zhao et al., 2005). It found that the error rate of high-frequency consonants (/p^h^, t^h^, f, tɕ^h^, tʂ, tʂ^h^, s, ʂ/), which have the largest amplitude in the spectrum over 3,000 Hz, was significantly higher than other consonants in older adults.

Mandarin consonants can be grouped into plosives (/p, p^h^, t, t^h^, k, k^h^/), fricatives (/ɕ, s, ʂ, f, x/), affricatives (/tɕ, tɕ^h^, ts, ts^h^, tʂ, tʂ^h^/), nasals (/m, n/), and A/LA (/l, ʐ/) according to the manner of articulation. In terms of the place of articulation, these consonants can be categorized into bilabials/labiodental (B/LD: /p, p^h^, m, f/), alveolars (/t, t^h^, n, l, ts, ts^h^, s/), palatals (/tɕ, tɕ^h^, ɕ/), velars (/k, k^h^, x/) and retroflexes (/tʂ, tʂ^h^, ʂ/). These consonants share some similarities with English but also have unique features. Unlike English which has voicing distinction in plosives and fricatives/affricatives, all these consonants in Mandarin are voiceless, and the plosives and affricatives have aspirated distinction. Besides, there are no consonant clusters in Mandarin and all consonants can only appear in the initial position, except for /n/. Moreover, it has been reported that the three palatal phonemes in Mandarin do not exist in English and are less common in other languages (Ladefoged and Maddieson, 1996). Another worth noting difference is that the three fricatives (/ɕ, s, ʂ/) share the same articulatory places as affricatives (/tɕ, tɕ^h^, ts, ts^h^, tʂ, tʂ^h^/), which may increase the perceptual difficulty due to the increased competition in a similar range.

From the acoustic perspective, voice onset time (VOT), formant transitions, and spectral moments were the most effective cues for recognizing consonants in most languages (Liberman et al., 1967; Forrest et al., 1988; Mitani et al., 2006; Li and Gu, 2015), with the values varied in different languages. For example, the VOT differences between voiced and voiceless consonants in English are around 20–30 ms, whereas the differences between aspirated and unaspirated consonants in Mandarin could be as large as 60 ms. It is reasonable to speculate that the larger VOT difference might facilitate the perception of the distinction of aspiration. Together, Mandarin consonants have many features that set them apart from other languages, both phonologically and acoustically. However, how aging affects consonant perception is still largely unknown.

### Older adults' consonant perception in less ideal conditions

There has been a large number of studies focused on older adults' speech perception in less ideal conditions, including noise, reverberation, time-compression, foreign-accent, or mixed degraded speech (Helfer and Huntley, 1991; Helfer, 1994; Gordon-Salant and Fitzgibbons, 2004; Ferguson et al., 2010; Alwan et al., 2011; Reinhart et al., 2016). These studies did report their worse performance than younger adults, but it also varied with conditions. For instance, reverberation and noise influenced consonant perception differently. Plosives were easier to understand in noise than in the reverberation plus noise condition and reverberation exerted more influence on the syllable-final consonants than syllable-initial consonants. It indicated that the underlying mechanisms for speech perception in these conditions might be different (Helfer, 1994). Reverberation and noise affected speech perception mainly through masking the temporal envelopes of original signals rather than changing any acoustic features of original speech (Fogerty et al., 2010), while time-compression and foreign accents mainly distort information such as the temporal cues inherent to natural speech.

Whispering is also a degraded speech condition. It might be difficult for older adults, but as far as we know, how whispering affects older adults' speech perception has yet to be explored. Whispered speech is characterized by a turbulent flow created by exhaled air passing through the opened glottis (Sundberg, 1994; Matsuda and Hideki, 1999). Recognizing whispered speech requires decoding these noisy and weak signals, which are therefore more demanding. The most prominent change in whispered speech is that the fundamental frequency is not available owing to the absence of vocal vibration, which resulted in a dramatic decrease in lexical tone perception (Gao, 2002; Jiao and Xu, 2019), intonation identification (Meyer-Eppler, 1956; Heeren and van Heuven, 2009; Heeren and Van Heuven, 2014; Zygis et al., 2017) and talker recognition (Lass et al., 1976; Fan and Hansen, 2011; Smith et al., 2017). Whispering also brings a shift of formants, especially for the first two formants, which leads to the perceptual difficulty for vowels (Kallail and Emanuel, 1984, 1985; Jovičić, 1998; Jiao and Xu, 2019).

Previous studies also demonstrated that whispering changed the acoustic features of consonants (Schwartz, 1972; Parnell et al., 1977; Jovičić and Šarić, 2008). It was found that the duration of whispered consonants was prolonged by about 10% on average and the prolongation of voiced consonants was greater than that of unvoiced. In the intensity domain, voiced consonants, that were nasals and A/LA, had reduced intensity over 25 dB but the intensity for unvoiced consonants remained unchanged. These acoustic changes brought perceptual difficulties, especially for the voiced-voiced distinction and the aspirated-unaspirated distinction (Dannenbring, 1980; Tartter, 1989; Mills, 2003; Meynadier et al., 2013). Our acoustic data (unpublished) on Mandarin consonants showed whispering led to an increased duration of unaspirated consonants but a decreased duration of their aspirated counterparts which narrowed the duration difference between them in whispered mode. Similarly, whispering lowered consonants' intensity, but increased the consonant-vowel intensity ratio (CVR), except for nasals. As a result of these changes in the acoustic properties, whispering may lead to heightened perceptual difficulties in Mandarin.

### The current study

As we mentioned above, a large number of studies have proved that aging was accompanied by high-frequency insensibility, temporal and spectral processing deficits, as well as higher thresholds of intensity and frequency, which may detrimentally affect consonant recognition. Whispering distorts the original speech owing to the lack of vocal vibration, which is different from the masked effects of noise or reverberation. Examining the effects of aging and whispering on Mandarin consonant identification might enrich our knowledge of speech perception in older adults, especially in challenging conditions. However, this question has yet to be investigated. To this end, we conducted an identification experiment with Mandarin consonants as stimuli. Older and younger listeners were required to recognize the 21 Mandarin consonants in phonated and whispered conditions. Consonants were mainly grouped by place and manner of articulation in statistical analyses. We hypothesized that older adults might show lower accuracy than younger adults, even in the phonated condition. Among the consonants, recognizing affricatives and fricatives that are mostly dependent on decoding high-frequency information might be most difficult for the elderly. In contrast, the identification of nasals and semivowels, which are based on formant information might be less affected. In terms of the aspirated-unaspirated distinction, it might be difficult as well considering the temporal processing deficit in older adults, but it is also possible that the difficulty might be compensated by the longer duration differences. In relation to whispering, we speculated that the identification of voiced consonants (nasals/A/LA), and aspirated-unaspirated distinction might be negatively affected in both age groups, but older adults will be more vulnerable to this condition.

## Methods

### Participants

Two groups of listeners, representing older and younger adults were recruited to participate in the current study. The older listener group consisted of 20 participants (16 females, 4 males) with ages ranging from 60 to 72 (Mean = 64.05, SD = 4.36). They were recruited in Shenzhen with the assistance of community managers. The younger listener group contained 20 students (8 females, 12 males) with an age range of 21–32 years old (Mean = 24.20, SD = 2.86). They were selected from the subject pool at Shenzhen Institute of Advanced Technology. Although the participants were recruited in Shenzhen, all the participants (younger and older adults) were from the northern China and spoke standard Mandarin. Furthermore, we have asked the participants to read the syllables on the hard copies before the formal experiment, which allowed us to make sure that all participants were able to distinguish the most confusing pairs, such as /ts-tʂ/, /ts^h^-tʂ^h^/, /s-ʂ/, and /n-l/. Due to the careful screening the dialect background of the participants, we assumed that the influence of sociolinguistic factors might have been kept at a lesser extent. Nobody reported neurological history or ear diseases. All participants were compensated for their participation. The experimental procedures were approved by the Human Subjects Ethics Committee of the Shenzhen Institutes of Advanced Technology, Chinese Academy of Science. Informed written consent was obtained from participants in compliance with the experiment protocols.

**Figure 1:**
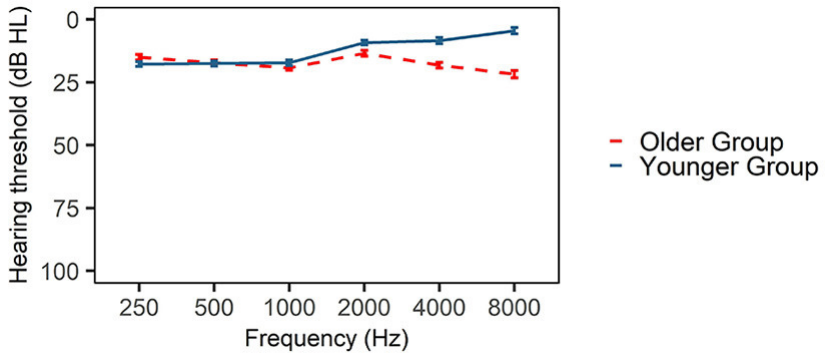
Pure tone thresholds at 250, 500, 1,000, 2,000, 4,000 and 8,000 Hz for the two listening groups.

A portable audiometer (GSI 18) was used to assess participants' hearing condition by air-conduction audiometry from 250 to 8,000 Hz. Given that the screening test was conducted in a quiet room rather than in a soundproof laboratory or a hospital, the average pure tone threshold lower than 25 dB rather than 20 dB was chosen to define as clinically hearing normal (Brandy, 2002). All pure-tone thresholds of younger adults were lower than 25 dB at all octave intervals between 250 Hz and 8,000 Hz, and their binaural threshold differences were lower than 15 dB at each frequency. Ten of the older adults had matched audiograms with younger adults. Their pure-tone thresholds were lower than 25 dB at all octave intervals between 250 and 8,000 Hz, and binaural threshold differences were lower than 15 dB at each frequency. The rest 10 of older adults had normal hearing below 4,000 Hz but mild hearing loss (<40 dB) at 8,000 Hz. The audiograms of two groups of listeners are shown in Figure 1. The hearing test took 10–15 min for each participant.

The cognitive abilities were tested with the Chinese version of Montreal Cognitive Assessment-Basic (MoCA-B) (https://www.mocatest.org/), which can be used for individuals with poor educational status. The test consists of 10 subtests: executive, memory, fluency, orientation, calculation, abstraction, delay recall, visual, naming, and attention. The scores of the older adults were all above 26 (Mean = 27.75, SD = 1.52), which indicate normal cognitive functioning. The cognitive screening lasted around 15 min.

### Stimuli

Mandarin consonants /p, p^h^, t, t^h^, k, k^h^, tɕ, tɕ^h^, ɕ, ts, ts^h^, s, tʂ, tʂ^h^, ʂ, f, x, m, n, l, ʐ/ were selected as target stimuli, and /a, i, u/ were used as the following vowels to ensure the diversity of the phonetic contexts. According to the classifications of Mandarin vowels, /a/ is an open vowel with the lowest tongue position, and /i/, /u/ are close vowels with the highest tongue position. The difference between /i/ and /u/ is the vowel backness with /i/ being a front vowel, while /u/ being a back vowel. The carrier lexical tones were high tone (Tone 1: 55). However, not all combinations of consonants, vowels, and lexical tones were legal in Mandarin. For example, the combinations of /tɕa55, tɕ^h^a55, ɕa55, ʐa55, tɕu55, tɕ^h^u55, ɕu55, ʐu55, ki55, k^h^i55, xi55, fi55, ʐi55/ do not exist in Mandarin. To rule out the possibility that the pseudoword might elicit inferior performance in older adults, these illegal syllables were replaced by most similar syllables which are real words in Mandarin: /tɕia55, tɕ^h^ia55, ɕia55, ʐa℧51 (Tone 4), tɕio℧55, tɕ^h^io℧55, ɕio℧55, ʐu51, keı214 (Tone 3), k^h^eı55, xei55, fei55, ʐi51/.

The original syllables were recorded with a female, speaking standard Mandarin, in a sound-attenuated laboratory. The target syllables were embedded in a sentence presented in both Chinese character and Pinyin through Powerpoint, for example, “这个音节是***aba***. zhè ge yin jié shì ***aba***” (This syllable is a***ba***). The speaker was given enough time to get familiar with the pronunciation of all stimuli. Each token was repeated at least three times to ensure the intelligibility of target syllables, giving rise to 6 blocks in total. Each block contained 21 target consonants. Phonated and whispered blocks were recorded alternately. For whispered sentences, the speaker was instructed to mimic whispering in a public library.

The first author selected the clearest syllable by observing the spectrogram and judging the intelligibility. Five younger listeners participated in a pilot study that had similar procedures to the formal study. The tokens with accuracy lower than 70% were replaced. Reevaluation was conducted until the tokens reached high intelligibility in younger adults. The target syllables were recorded and segmented at 44,100 Hz and 24 bits. Acoustic analyses were conducted for the selected phonated syllables (Mean duration = 621 ms, SD = 113 ms; Mean intensity = 57.22 dB, SD = 6.13 dB) and whispered syllables (Mean duration = 588 ms, SD = 111 ms; Mean intensity = 51.04 dB, SD = 7.05 dB). The selected syllables were then scaled to a mean duration of 600 ms at 60 dB by Praat. All the stimuli were presented at 60 dB sound pressure level (SPL) measured by sound pressure meter (Rion NL-21).

### Procedure

The procedure was a 21-alternative forced-choice identification task. Psychological software E-prime 3.0 was used to present stimuli and collect data. The response syllables were displayed on a computer monitor in Pinyin forms in six rows and four columns. The syllables in each row shared a similar place of articulation, for example, the first row contained bilabials and labiodental, the second row contained alveolars, and the third row contained velars. The auditory stimuli were randomly presented to listeners *via* Sennheiser 280 headphones binaurally. Participants were asked to identify the consonant by selecting one of the choices presented simultaneously on the screen as correctly and quickly as possible. Whispered and phonated stimuli were presented in separate blocks. There were six blocks that varied in articulatory modes and vowels. Each block consisted of 21 trials, each of which was repeated three times. The order of the blocks was counterbalanced. Participants could take a break after each block or after 30 trials within a block.

Listeners, especially older adults, were provided enough time to familiarize themselves with the stimuli and procedures. This was to ensure that consonant identification was affected by aging and articulatory conditions rather than other factors. In the familiarization stage, as has been described briefly above, participants were first asked to read out the 63 syllables (the combinations of 21 consonants and 3 vowels) printed on the hard copies. Participants who could not read consonants correctly had to be excluded. Note that to minimize the influence of Pinyin proficiency on the perceptual identification results, the syllables were presented in Pinyin format, which were identical to the materials used in the formal test. Only those who can recognize Pinyin accurately were invited to the formal experiments. Secondly, listeners were required to complete two practice blocks (a phonated condition and a whispered condition) with feedback given for each trial, including accuracy and the correct answer. The auditory stimuli used in practice sessions were recorded by another female speaker. The participants were asked to redo the practice if their scores were lower than 50% in the phonated block. They would not be invited to the formal test if their accuracy was still lower than 50%. Nine older adults were excluded during this process.

### Data analysis

Identification accuracy and reaction time (RT) were analyzed with a series of generalized linear mixed-effects models (for accuracy) and linear mixed-effects models (for RT). For accuracy analyses, trials with response times beyond the 500–7,500 ms were excluded as a result of accidental key pressing or lapses in attention. A total of 819 trials were excluded, accounting for 5.4% of the data. For RT, incorrect responses (26.11%) and responses over 2 SDs from the mean (3.88%) were excluded. The original data were then log-transformed for statistical analyses. The statistical analysis aims to elucidate consonant identification in two listener groups and in different places and manners of articulation. Generalized linear mixed-effects models were first fitted with group (younger adults, older adults), condition (phonated speech, whispered speech), and place (B/LD, alveolar, palatal, velar, retroflex), and their interactions as fixed effects, and subject was included as a random effect. Additional generalized mixed-effects models were then fitted with group, condition, and manner (plosive, fricative, affricative, nasal, A/LA), and their interactions as fixed effects, and with subject as a random effect. Further, more generalized linear mixed-effects models were fitted to investigate the identification differences within plosives, fricatives, and affricatives. Finally, to rule out the influence of high-frequency hearing loss (at 8,000 Hz), a series of generalized linear mixed-effects models were conducted to test whether there are differences between two older adults with normal hearing and mild high-frequency hearing loss. When there were significant main effects or interactions, Bonferroni *post-hoc* tests were applied for pairwise comparisons. The data analyses were performed with R (Version 4.0.5), using the *lme4* package (Bates et al., 2015) and *emmeans* package (Lenth, 2018).

## Results

**Figure 2:**
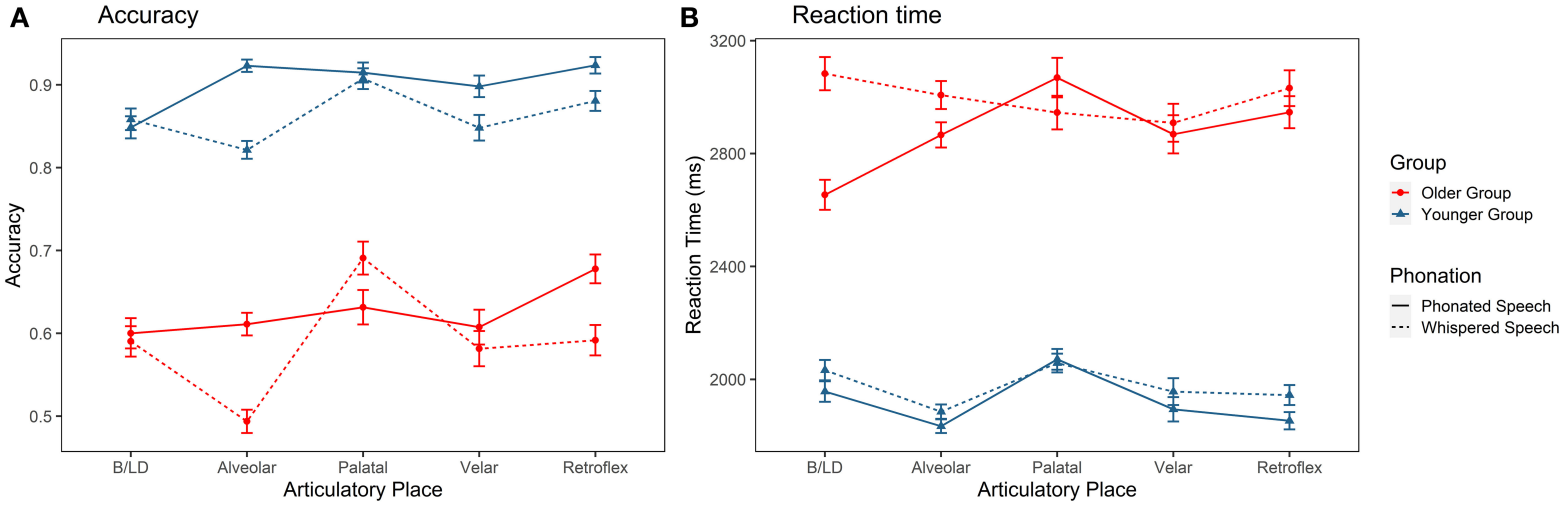
Identification accuracy **(A)** and reaction time **(B)** of the Mandarin consonants grouped by place of articulation in older and younger listeners. B/LD, bilabial and labiodental.

As shown in Figure 2A, the identification accuracy of consonants with different places of articulation differs among the two listener groups and articulatory modes. There were significant main effects of group [$\chi_{\left(1\right)}^{2}$ = 73.909, *p* < 0.001], condition [$\chi_{\left(1\right)}^{2}$ = 46.123, *p* < 0.001], and place [$\chi_{\left(4\right)}^{2}$ = 63.639, *p* < 0.001]. The interaction effects of group × place [$\chi_{\left(4\right)}^{2}$ = 4.951, *p* < 0.01], condition × group [$\chi_{\left(1\right)}^{2}$ = 7.016, *p* < 0.001], and condition × place [$\chi_{\left(4\right)}^{2}$ = 60.494, *p* < 0.01] were also significant. Three-way interaction was not significant (*p* > 0.05). *Post-hoc* tests indicated that older listeners identified B/LD, alveolars, palatals, velars, and retroflexes less accurately than younger adults (*ps* < 0.001). Furthermore, whispering lowered the accuracy of alveolars, retroflexes and velars (*ps* < 0.05]. Regarding RTs (Figure 2B), there were significant main effects of group [$\chi_{\left(1\right)}^{2}$ = 44.124, *p* < 0.001], condition [$\chi_{\left(1\right)}^{2}$ = 33.441, *p* < 0.001] and place [$\chi_{\left(4\right)}^{2}$ = 61.944, *p* < 0.001]. The interaction effects of group × place [$\chi_{\left(3\right)}^{2}$ = 44.300, *p* < 0.001], and group × condition × place [$\chi_{\left(9\right)}^{2}$ = 57.368, *p* < 0.001] were also significant. The *post-hoc* tests showed that older adults employed longer time in recognizing all consonants than younger adults under both phonated and whispered conditions. Among different consonants, the RT used in recognizing whispered labials was much longer than other types of consonants for older adults.

**Figure 3:**
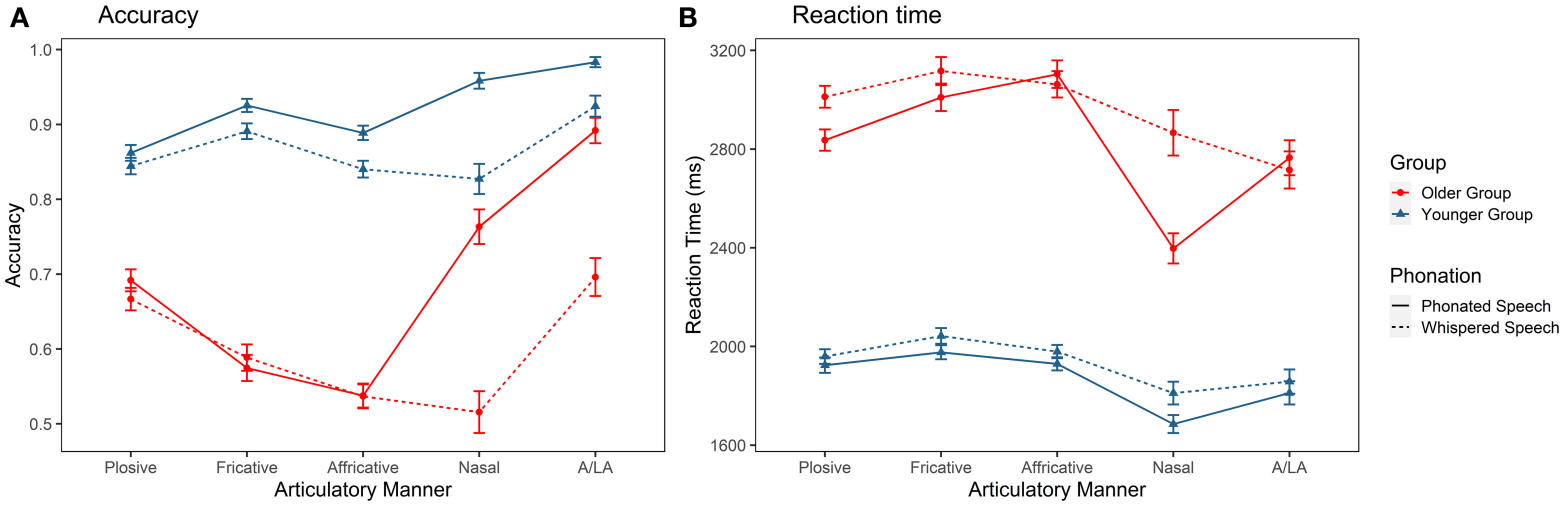
Identification accuracy **(A)** and reaction time **(B)** of the Mandarin consonants grouped by manner of articulation in older and younger listeners. A/LA, approximant and lateral approximant.

Figure 3A demonstrates the identification accuracy of consonants with different manners of articulation in the two listener groups and articulatory modes. There were significant main effects of group [$\chi_{\left(1\right)}^{2}$ = 70.584, *p* < 0.001], condition [$\chi_{\left(1\right)}^{2}$ = 41.720, *p* < 0.001], and manner [$\chi_{\left(4\right)}^{2}$ = 156.307, *p* < 0.001]. The interaction effects of group × manner [$\chi_{\left(4\right)}^{2}$ = 76.300, *p* < 0.001], group × condition [$\chi_{\left(1\right)}^{2}$ = 9.655, *p* < 0.001], and condition × manner [$\chi_{\left(4\right)}^{2}$ = 94.525, *p* <0.001] were also significant. *Post-hoc* tests revealed that older adults had lower recognition accuracy than younger adults in two conditions (*ps* <0.001). In addition, the *post-hoc* tests also indicated that two listener groups showed different perceptual patterns. For younger adults, the accuracy for A/LA was highest among all types of consonants (*ps* < 0.001) and the accuracy for nasals was higher than plosives and affricatives (*ps* < 0.05). As for fricatives and affricatives, affricatives seemed more difficult than fricatives (*p* < 0.001). For older adults, similar to younger adults, the accuracy for A/LA was highest (*ps* < 0.001). The accuracy for nasals was also higher than fricatives and affricatives (*ps* < 0.05). As for fricatives and affricatives, affricatives were also more challenging than fricatives (*p* < 0.001). The most notable difference between the two listener groups was that older adults performed less accurately on the identification of affricatives and fricatives than other types of consonants (*ps* < 0.001). In terms of the effect of whispering, it lowered the accuracies of nasals and A/LA for older adults (*ps* < 0.001). Affricative identification was also negatively affected (*ps* < 0.001) in addition to nasals and A/LA for younger adults. In terms of RTs (Figure 3B), there was a main effect of group [$\chi_{\left(1\right)}^{2}$ = 44.124, *p* < 0.001], with the RT longer in the older listener group. There was also a main effect of condition [$\chi_{\left(1\right)}^{2}$ = 33.441, *p* < 0.001], in that RT used in recognizing whispered consonants was significantly longer. Lastly, there was a main effect of manner of articulation [$\chi_{\left(4\right)}^{2}$ = 200.310, *p* < 0.001], showing that the RT used in recognizing nasals was shortest. No other effects were significant.

**Figure 4:**
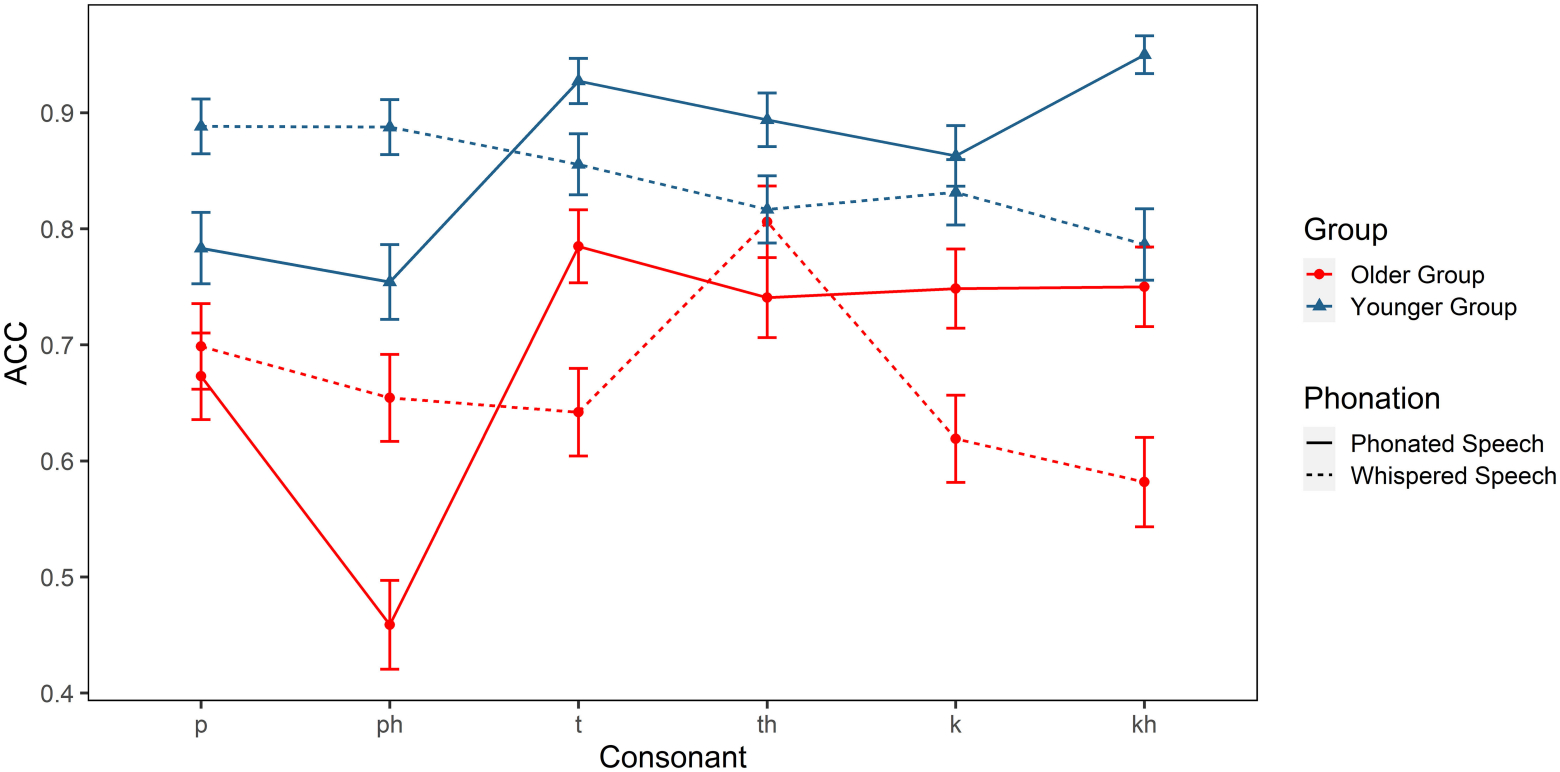
Identification accuracy of the Mandarin plosives (/p, p^h^, t, t^h^, k, k^h^/) in older and younger listeners.

**Figure 5:**
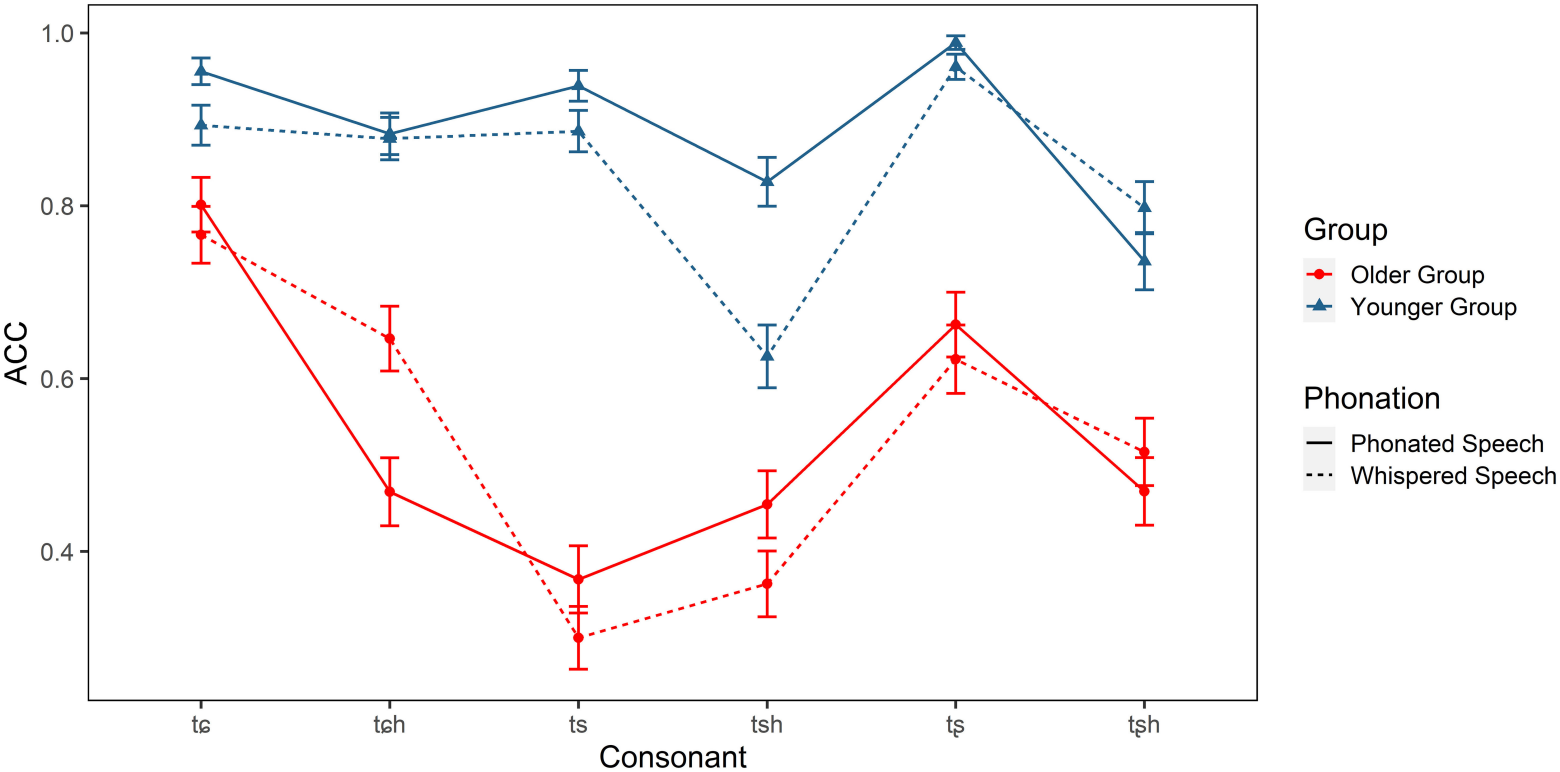
Identification accuracy of the Mandarin affricates (/tɕ, tɕ^h^, ts, ts^h^, tʂ, tʂ^h^/) in older and younger listeners.

**Figure 6:**
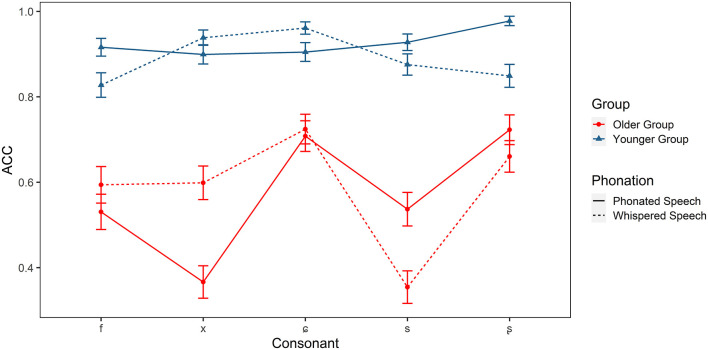
Identification accuracy of the Mandarin fricatives (/f, x, ɕ, s, ʂ/) in older and younger listeners.

In order to examine the perceptual patterns in a more detailed manner, linear mixed effects models were further constructed for plosives, affricatives, and fricatives separately. Group, condition, and consonant (plosives: /p, p^h^, t, t^h^, k, k^h^/; affricatives: /tɕ, tɕ^h^, ts, ts^h^, tʂ, tʂ^h^/; fricatives: /f, x, ɕ, s, ʂ/), and their interactions were treated as fixed effects and subject was treated as a random effect. Figures 4–6 show the results.

For plosives, there were significant main effects of group [$\chi_{\left(1\right)}^{2}$ = 21.551, *p* < 0.001] and consonant [$\chi_{\left(5\right)}^{2}$ = 39.504, *p* < 0.001]. The interaction effects of group × consonant [$\chi_{\left(5\right)}^{2}$ = 14.447, *p* < 0.05], condition × consonant [$\chi_{\left(5\right)}^{2}$ = 75.022, *p* < 0.05], and group × condition × consonant [$\chi_{\left(5\right)}^{2}$ = 13.253, *p* < 0.05] were also significant. *Post hoc* analyses showed that the accuracy for /p^h^, k^h^/ was significantly lower in older adutls (*ps* < 0.05) but the accuracy for /p, t, k/ was comparable between the two groups (*ps* > 0.05) in phonated condition. In whispered condition, older adults identify /t, k, p^h^/ less accurately than younger adults (*ps* < 0.001). As for the effect of whispering, it lowered the identification accuracy of /k^h^/ in two groups (*ps* < 0.05) but it facilitated the recognition of /p^h^/ for both groups (*ps* < 0.05) which was contrary to our expectations.

For affricatives, there were significant main effects of group [$\chi_{\left(1\right)}^{2}$ = 71.827, *p* < 0.001] and consonant [$\chi_{\left(5\right)}^{2}$ = 213.502, *p* < 0.001]. The interaction effects of group × condition [$\chi_{\left(1\right)}^{2}$ = 7.734, *p* < 0.01], group × consonant [$\chi_{\left(5\right)}^{2}$ = 70.927, *p* < 0.001], and condition × consonant [$\chi_{\left(5\right)}^{2}$ = 34.056, *p* < 0.001] were also significant. *Post-hoc* tests indicated that older adults identified all affricatives less accurately than younger adults in both conditions (*ps* < 0.001). Moreover, whispering resulted in a lower accuracy score of /ts, ts^h^, tɕ/, (*p* < 0.05), but a higher score of /tɕ^h^/.

For fricatives, there were significant main effects of group [$\chi_{\left(1\right)}^{2}$ = 70.886, *p* < 0.001] and consonant [$\chi_{\left(4\right)}^{2}$ = 60.053, *p* < 0.001]. The interaction effects of group × condition [$\chi_{\left(1\right)}^{2}$ = 4.651, *p* < 0.05], group × consonant [$\chi_{\left(4\right)}^{2}$ = 23.084, *p* < 0.001], condition × consonant [$\chi_{\left(4\right)}^{2}$ = 44.665, *p* < 0.001], and group × condition × consonant [$\chi_{\left(4\right)}^{2}$ = 16.520, *p* < 0.01] were also significant. *Post-hoc* analyses showed that older adults had difficulties in recognizing all fricatives in two conditions (*ps* < 0.01). Fricatives /f, x, s/ were more challenging for older adults (*ps* < 0.01) compared with /ɕ, ʂ/ in the phonated condition, while /s/ was most difficult to be identified in whispered condition (*ps* < 0.001). Whispering especially lowered the accuracy of /ʂ/ for younger adults and /s/ for older adults. However, the accuracy of /x/ was increased in the whispered condition in older adults.

Lastly, to rule out the possibility that hearing loss may also have played a role in the results, additional generalized linear mixed-effects models were fitted. It revealed that there were no significant differences between the two groups of older adults (with normal hearing and mild hearing loss in high-frequency) for all performances mentioned above (*ps* > 0.05).

We also analyzed the perceptual error patterns. Table 1 demonstrates the most frequent confusion pairs and their error percentage for younger and older adults in two articulatory modes. In phonated condition, younger adults' confusion was within bilabial plosives (/p-t/, /p^h^-t^h^/), aspirated affricatives (/tʂ^h^-ts^h^/), as well as affricatives and fricatives with the same place of articulation (/ts-s/, /tɕ^h^-ɕ/). Compared with younger adults, older adults demonstrated more confusing pairs. They were more confused about aspirated bilabial plosives /p^h^-t^h^/. In addition, pairs of affricatives and fricatives distinguished by high-frequency information seemed confusing to them too. For example, the most frequent confusion pairs of /ts-tʂ/, /s-ʂ/, /ts^h^-tʂ^h^/ and /tɕ^h^-ɕ/ were observed in older adults. In whispered condition, both listener groups exhibited more confusion pairs. Differentiating aspirated and unaspirated consonants, such as the pair /p-p^h^/, /t-t^h^/, /k-k^h^/, /tɕ-tɕ^h^/, /tʂ-tʂ^h^/ was challenging for older adults, implying that processing temporal cues was more difficult in whispered condition for them. In addition, consonants with high-frequency information were also difficult to distinguish for older adults too, especially for /ts, ts^h^, s, tʂ, tʂ^h^, ʂ/. Lastly, distinguishing voiced consonants, including nasals (/m-n/) and A/LA (/l-ʐ/) became more challenging for both groups.

**Table 1:** Most frequently confused pairs and their error percentage for two groups in two articulatory modes.

**Phonated speech**				**Whispered speech**					
**Younger group**		**Older group**		**Younger group**		**Older group**			
p-t	20%	p-t	12.78%	t-k	10.56%	p-t	10%	p-p^h^	14.44%
p^h^-t^h^	20%	p^h^-t^h^	36%	t^h^-k^h^	8.89%	p^h^-p	17.50%	p^h^-t^h^	11.67%
tʂ^h^-ts^h^	18.33%	k^h^-t^h^	16.67%	k-k^h^	14.44%	t-p	10%	t-t^h^	11.67%
ts-s	11.11%	x-t^h^	15.56%	k^h^-k	18.89%	k-k^h^	31.11%		
tɕ^h^-ɕ	10%	x-k^h^	13.89%	ts-tʂ	8.33%	k^h^-k	31.67%		
		x-p^h^	11.67%	ts^h^-s	23.89%	tɕ-tɕ^h^	10.56%		
		tɕ^h^-ɕ	39%	ts^h^-ts	8.33%	tɕ^h^-tɕ	13.89%	tɕ^h^-ɕ	14.44%
		ɕ-tɕ	20.56%	tʂ^h^-tʂ	15.56%	ɕ-tɕ^h^	8.33%		
		ts-tɕ	25.56%	ɕ-ʐ	11.67%	ts-tʂ	37.22%		
		ts-ʐ	13.89%	m-n	8.33%	ts^h^-tʂ^h^	21.11%	ts^h^-s	18.89%
		ts^h^-tʂ^h^	24.24%	ʐ-ʂ	7.22%	s-ʂ	19.44%	s-tʂ^h^	11.67%
		s-ʂ	25.56%			tʂ-ts	22.22%	tʂ-tʂ^h^	11.11%
		tʂ-ts	23.89%			tʂ^h^-tʂ	20%	tʂ^h^-ts	16.67%
		tʂ^h^-ts^h^	18.33%			ʂ-s	23.89%		
		ʂ-s	15.56%			f-p^h^	11.67%		
		n-m	21.11%			m-n	10%		
						n-m	10%	n-ʐ	10%
						l-n	10%		
						ʐ-ʂ	21.67%		

## Discussion

There are many potential factors contributing to consonant identification, including listeners' speech processing ability and cognition ability, acoustic characteristics and the phonological structure of consonants, whether it is presented in a degraded mode, and the difficulty of tasks etc. The current study aims to investigate the effects of aging and whispering on Mandarin consonant identification. Our hypothesis was that cognitive decline and aging-related speech processing deficits, such as the insensitivity to high-frequency information and the deteriorating temporal and spectral processing ability, would adversely affect older adults' consonant identification in normal condition. Moreover, whispering would further worsen the consonant identification in older adults as the acoustic differences became less prominent in whispered speech. It is noteworthy that in the current study, the intensity of phonated and whispered syllables was scaled to the same level (60 dB) to ensure speech audibility, which might reduce the intensity differences between phonated and whispered consonants. However, this manipulation does not affect the relative intensity levels for the consonants. For instance, the ratio between intensity of the consonant and the whole syllable was kept unchanged and the differences among the consonants were also not affected. Therefore, we believe the manipulation on intensity levels may have limited effects on cues for the perception of place/manners of the consonants in the current study.

### The effect of aging on consonant perception

For older adults, affricatives and fricatives were more challenging, followed by the plosives and nasals, while A/LA were relatively well-recognized. This is largely consistent with previous findings which showed the information of stop, place, and friction characteristics decreased more than nasality, liquid/glide quality features for older adults in quiet conditions (Gelfand et al., 1985; Gordon-Salant, 1986). Consonant recognition is a complex process involving decoding and integrating various acoustic cues and the dependent cues varies among consonants. Thus, the uneven consonant perception might be attributed to different acoustic cues and older adults' decoding abilities.

In Mandarin, successfully identifying affricatives is related to the discrimination of the place of articulation and aspiration. Aspiration discrimination largely relies on listeners' superior temporal processing ability to decode VOT information, and recognition of the place of articulation depends on the sensitivity to spectral features in high frequency. The results of perceptual error patterns revealed that the most confusing pairs in older adults were /tɕ^h^-ɕ/, /ɕ-tɕ/, /ts-tʂ/, /ts^h^-tʂ^h^/, /s-ʂ/ rather than /tɕ-tɕ^h^/, /ts-ts^h^/, /tʂ-tʂ^h^/. This phenomenon indicated that the identification difficulties of affricatives and fricatives in older adults were largely attributed to their decreased high-frequency processing ability to recognize articulatory places, rather than the temporal processing deficit in distinguishing aspiration. The relatively high accuracy to distinguish aspirated-unaspirated distinction may be due to the specific acoustic features of affricatives and fricatives in Mandarin. As we mentioned before, the duration differences between aspirated and unaspirated consonants were larger than 60 ms, which may facilitate listeners' perception. Thus, although older adults are considered to suffer from temporal processing deficits, it didn't severely affect the aspiration distinction in Mandarin.

Although identifying fricatives /f/ and /x/ also depends on high-frequency information, the matrix analyses indicated that the processing deficit in older adults could not adequately explain their perceptual difficulties, as these two fricatives were often confused with aspirated plosives, /p^h^, t^h^, k^h^/, except for other affricatives and fricatives. Other features of these two fricatives might account for the difficulties. The first possible feature might be their lower intensity relative to other fricatives. Some studies found that intensity had less direct impacts on consonant perception (Zeng and Turner, 1990; Scharenborg and Janse, 2012), but other studies showed that intensity had interactive effects with other factors in phoneme perception. For example, Nábělek et al. (1996) found the transition with high intensity facilitated the perception of diphthongs in older adults, especially in less ideal conditions. Another possible reason is that the energy distribution of these two fricatives is homogeneous throughout the spectrum which makes them perceptually confusing with other aspirated plosives. The deteriorated ability to process high-frequency cues combined with insensitivity to low intensity information of older adults makes these fricatives difficult to be distinguished.

For plosives, older adults' ability to categorize unaspirated-aspirated distinction was largely preserved, which was consistent with the results of affricatives. However, they encountered more difficulties in identifying the place of articulation in plosives. Formant transitions, especially for the second and third formants were crucial cues for the recognition of articulatory places in plosives (Liberman et al., 1952; Tsui and Ciocca, 2000; Alwan et al., 2011). Further detailed analysis revealed that the place distinction in aspirated plosives (/p^h^, k^h^/), rather than unaspirated ones (p, t, k), was particularly challenging. This difficulty might be attributed to the lower clarity of formant transitions in aspirated plosives and older adults' impoverished ability to process formant transitions. The formant transitions of voiceless plosives are basically completed before the voice onset owing to the relatively long voice onset time in English (Stevens and Klatt, 1974). The case might be similar in Mandarin in that the formant transitions of aspirated plosives are completed before the voice onset. As a result, the formant transitions were embedded in the noise part which further lowered the clarity. Moreover, older adults are proved less efficient in dealing with formant transition information. Several categorical perception experiments have indicated that older adults cannot categorize the places of articulation in plosives well solely based on the dynamic formant transition information (Dorman et al., 1985; Plyler and Hedrick, 2002; Harkrider et al., 2005). This deficit was further supported by neural evidence showing that the second-formant onset frequency elicited reduced subcortical amplitude in older adults (Rishiq et al., 2020). Our findings provided additional evidence to the formant transition perception deficit in older adults.

It is worth noting that the effect of aging on consonant identification reported here was not only found on the response accuracy but also on the processing speed. Generally, we found older adults employed longer time in the consonant recognition. Combined with the overall significantly lower accuracy, the RT data can be considered as a sensitive measure to capture the processing disadvantage of older participants, suggesting that older adults were less capable in this consonant categorization task. This finding of age-related processing speed decline is also consistent with previous studies showing that older adults are associated with general slower speed in both speech and cognitive tasks (Dey and Sommers, 2015).

### The effect of whispering on consonant perception

Findings of the current study revealed that the identification of nasals and A/LA was compromised in whispered condition in both groups, especially for older adults, whereas the accuracy of voiceless consonants was less affected. It was also found that RT in the whispered condition was significantly longer for most consonants. The absence of vocal fold vibration in whispering turns voiced consonants to voiceless. The turbulent flow in whispering also distorted the resonance which results in degraded formant information. Furthermore, whispering lowered the intensity of consonants (Jovičić and Šarić, 2008). Several studies have demonstrated that reduced-intensity compromised speech perception (Gordon-Salant, 1986; Nábělek et al., 1996; Kennedy et al., 1998), especially in less ideal conditions. For example, Nábělek et al. (1996) found that the reduction of intensity ranging between 1 and 15 dB affected the diphthong perceptions, particularly for listeners with hearing loss and in noise and reverberation conditions. We speculated that the distorted formants, low intensity collectively lead to the difficulty of voiced consonant recognition and longer RT in whispered speech. Older adults' poor performance further indicated that they may have difficulty processing degraded signals, especially the voiced information, at lower intensity.

Analyses of the matrices showed that the error pattern in the two articulatory modes was different, particularly for plosives and affricatives. In phonated condition, most of the difficulties were differentiating articulatory places, while in whispered condition, distinguishing aspirated from unaspirated consonants became also challenging, especially for older adults. Our acoustic data (unpublished) showed that whispering led to the decreased frication duration of aspirated consonants and increased frication duration of unaspirated consonants, which narrowed the duration differences between aspirated and unaspirated consonants. It is possible that this reduced duration difference, together with temporal processing deficit in older adults, leads to more errors.

Contrary to our expectation, whispering increased the identification accuracy of /p^h^/ for both groups, and /x, tɕ^h^/ for older adults. As mentioned above, /x/ was one of the most difficult fricatives for older adults in phonated condition, owing to the lower intensity and homogeneous energy distribution. The increased intensity in whispered condition and intensity manipulation of stimuli may also have played a role here.

Whispering is one of the challenging listening conditions in our daily life. Whether it influences speech perception differently compared to other degraded speech is less studied in the literature. In a previous study, Helfer (1994) investigated the English consonants perception (/b, d, g, p. t, k, f, v, s, z, θ, ð, m, n/) in three less ideal conditions, that is noise, reverberation, and noise plus reverberation conditions. Comparing our findings with it, we found that whispering led to different perceptual error patterns. Firstly, whispered speech detrimentally affected nasals and A/LA while the accuracy of nasals was well-maintained in all the three degraded conditions in the previous study. Secondly, whispering resulted in new error patterns while the error patterns in other three less ideal conditions were similar to the normal condition. For example, new error patterns (/k-k^h^/, /ts-ts^h^/, /tʂ-tʂ^h^/) emerged in whispered condition only. On the contrary, distinguishing articulatory places (/b-g/, /p-t-k/) was most difficult in both normal and other three degraded conditions. The possible reason is that speech perception difficulties in noise and reverberation are caused by these additional interferences (i.e., noise) to target phonemes, while the difficulties in whispering are attributed to the changed quality of targets. Lastly, the previous study found that some fricatives with weaker energy were more likely to be masked by noise while relatively resistant to reverberation. In contrast, our study found that certain fricative, i.e., /x/, became easier in whispering. These results suggested that the underlying mechanism under whispered speech perception might be different from other degraded conditions, owing to it is special phonation nature.

### Limitations

In the current study, to make the two conditions comparable, the phonated and whispered syllables were scaled to having the same duration and intensity. Some of their original/natural cues and the difference between the two articulatory modes may have been changed due to these manipulations. Stimuli without any manipulation could be used to retest the effects of whispering on consonant identification in future studies. Another question is that tokens from only one female speaker were used as stimuli. Previous studies of consonant perception showed response to the same phoneme could be variable due to various factors, such as acoustic differences in speech, within- and across- speakers/listeners (Cutler et al., 2004; Toscanoa and Allen, 2014; Zaar and Dau, 2015). Further studies may increase the stimulus complexity by including more talkers to better understand the effects of aging and whispering on consonant perception in Mandarin. Lastly, we have administered cognitive tests to exclude those with cognition impairment. We have also provided adequate practice sessions and did not set a response time limit in order to reduce cognitive load. However, the 21-alternative forced-choice task might still be challenging for older adults. Therefore, declined cognitive ability could not be completely ruled out as one of the possible reasons older adults' lower accuracy and longer RT in consonant identification.

## Conclusion

The current study aims to explore whether and how aging and whispering affect consonant identification. Considering older adults' cognition declining, deteriorated sensitivity to high-frequency information, poor ability to process temporal and spectral cues and other listening deficits documented in previous literature, we hypothesized that the consonant identification ability in the aging population might decline, even in the quiet condition. The results confirmed our hypothesis and revealed that the difficulty in different consonants was uneven. The recognition of affricatives and fricatives based on decoding high-frequency information is most challenging in the elderly population even for those with normal hearing condition. Identifying the place of articulation in affricatives and plosives was more difficult as well. We also hypothesized that whispering may further affect consonant identification, particularly for older adults. The results showed that the degraded formant information and lower intensity in whispering adversely affected the identification of voiced consonants (nasals and L/LA). Moreover, the duration differences between aspirated and unaspirated consonants decreased in the whispered condition which resulted in difficulties in distinguishing these two types of consonants. Contrary to our expectations, whispering facilitated the identification of certain consonants, such as /p^h^, tɕ^h^, x/. Our study increased the understanding of how aging affects speech perception in Mandarin which has unique phonological and acoustic features. Findings on whispered speech perception also enriched our knowledge of speech perception under different less ideal conditions, which in turn may assist us in developing effective speech rehabilitation strategies for older adults.

## Data availability statement

The datasets presented in this study can be found in online repositories. The names of the repository/repositories and accession number(s) can be found below: https://osf.io/rhwqv/.

## Ethics statement

The studies involving human participants were reviewed and approved by Human Subjects Ethics Committee of the Shenzhen Institutes of Advanced Technology, Chinese Academy of Science. The patients/participants provided their written informed consent to participate in this study.

## Author contributions

MX and JS contributed to conception and design of the study, and prepared the manuscript. MX collected data and performed the statistical analysis. All authors contributed to manuscript revision, read, and approved the submitted version.

## Funding

This work was supported by grants from the National Natural Science Foundation of China (NSFC: 11904381), the National Key R&D Program of China (2020YFC2004100), and the Start-up Grant from Hong Kong Baptist University (162646).

## Conflict of interest

The authors declare that the research was conducted in the absence of any commercial or financial relationships that could be construed as a potential conflict of interest.

## Publisher's note

All claims expressed in this article are solely those of the authors and do not necessarily represent those of their affiliated organizations, or those of the publisher, the editors and the reviewers. Any product that may be evaluated in this article, or claim that may be made by its manufacturer, is not guaranteed or endorsed by the publisher.
